# Climatic variability at Gangtok and Tadong weather observatories in Sikkim, India, during 1961–2017

**DOI:** 10.1038/s41598-020-71163-y

**Published:** 2020-09-16

**Authors:** Parvendra Kumar, Milap Chand Sharma, Rakesh Saini, Girish Kumar Singh

**Affiliations:** 1 Department of General and Applied Geography, Dr. Harisingh Gour Central University, Sagar, Madhya Pradesh 470003 India; 2 Centre for the Study of Regional Development, Jawaharlal Nehru University, New Delhi, 110067 India; 3 Department of Computer Science, Dr. Harisingh Gour Central University, Sagar, Madhya Pradesh 470003 India

**Keywords:** Climate change, Climate sciences

## Abstract

The present study documents the long-term trends in the temperature and precipitation of a poorly represented region, the Sikkim, eastern Himalaya using the Mann–Kendall non-parametric test and the Sen’s slope estimator. Additionally, the normal distribution curves and Cusum charts have been used to identify the shifts in extreme events and to detect the points of change in the climatic data series for robust analysis. The minimum temperatures recorded a positive trend in Gangtok (0.036 ˚C year−^1^ from 1961 to 2017) as well as in Tadong (0.065 ˚C year−^1^ from 1981 to 2010) stations, while the maximum temperatures showed no trend in Tadong station from 1981 to 2010 which is consistent with the trend in Gangtok station for the overlapped period. However, it was negative for the overall assessed period (− 0.027 ˚C year−^1^ from 1961 to 2017) in Gangtok. The average temperatures in Gangtok recorded no trend whereas a positive trend (0.035 ˚C year−^1^ from 1981 to 2010) was observed at Tadong station. A similar positive trend in the average temperatures has been detected at Gangtok also for the overlapped period. Accelerated warming was noticed during the last two decades with an increase in the probability of extreme events of temperatures (minimum, maximum, average) at the higher end. Precipitation was found to be more variable across the observed period and suggested no trend in the study area.

## Introduction

Climate change is the most sensitive and disputed subject in recent times. Studies have suggested warming and increasing pluvial conditions since the twentieth century at the global level^1–7^ and wider impacts of the global climatic changes on the existing eco-cultural landscape^8^. Nevertheless, discrepancies exist, particularly in understanding the pattern and trend of climate at the local and regional level which can eventually be improved by increasing research in the under-represented regions of the world. Key issues like restricted accessibility, remoteness and unavailability of instrumental climatic data have led to alpine environments been under-represented in the global climatic predictions. As of today, a little is known about the existing climatic scenario in the regions located at different latitudes and relief. It is noteworthy that the alpine environments encompass diverse geo-environmental settings and are most sensitive and vulnerable to the diverse impacts of climate change^9^. Therefore, intensive studies need to be carried out in these regions.

In this context, climatic studies in different parts of the Himalayas (comprising of immense relief and transition of climates) have become a crucial and utmost need as large population exists in the forefield of these mountains. It is widely acknowledged that poor economies of the world are more vulnerable to climate changes with serious implications for billions of people^10,11^ which further strengthens the reason for more studies in the region. However, the paucity of the long-term instrumental climatic data in the Himalayan region has limited the scientific observations. The available limited climatic studies, suggesting warming trends at diverse rates ^10,12–14^, have largely focused on the western and central Himalayas. With regard to the analysis of the precipitation as well, the central and NW Himalaya dominates the results^15–17^. Therefore, it clearly envisages that there is certainly a need to have research from the underrepresented regions including the eastern Himalaya, which eventually pave the way for the representation of the complex climatic system of the region.

Sikkim Himalaya, a part of the eastern Himalaya, which is influenced by the tri-junction of climatic systems largely dominated by the SW monsoon and receive limited winter rain from the Mediterranean westerly, and North-east monsoon, provides a vantage location to understand the complex responses of the climatic changes. The Sikkim Himalaya is also known to be a part of the biodiversity hotspot. The major population of the Sikkim Himalaya is rural (∼ 75%) and economically dependent on climatically sensitive sectors such as agriculture and tourism for its livelihood^18^. Besides, the area also contains more than 100 glaciers which are the lifeline to the region. Therefore, the climatic study of this region is not only important to understand the physical atmospheric system but is also crucial to assess the influence of any climatic change on the biophysical and socio-economic setup of this region. However, understanding the region’s climate is still underrated as only a few studies have been carried out on the Sikkim Himalayas. Rahman et al. (2008) and Seetharam (2008) have presented a primary discussion about mean monthly temperatures and precipitation on a decadal scale in Sikkim Himalaya^19,20^. Sharma and Shrestha (2016) analyzed the perceptions on climate change among people of Sikkim and tried to validate the results with trends of instrumental data of temperature and precipitation between 1978 and 2009^18^. However, they did not provide any information about the level of understanding of respondents regarding climate and weather systems. The present study, therefore, focuses on Sikkim Himalaya to fill the gaps in understanding of the climatic changes in detail and its impact on the region. The paper further attempts to analyze the climatic trends in the region from 1961 to 2017 (longest period than any other study have reported from the region). The study also investigates the behavior of monthly extreme events of temperature and precipitation and attempts to pin-point the year of change in the trend of climatic data.

**Figure 1:**
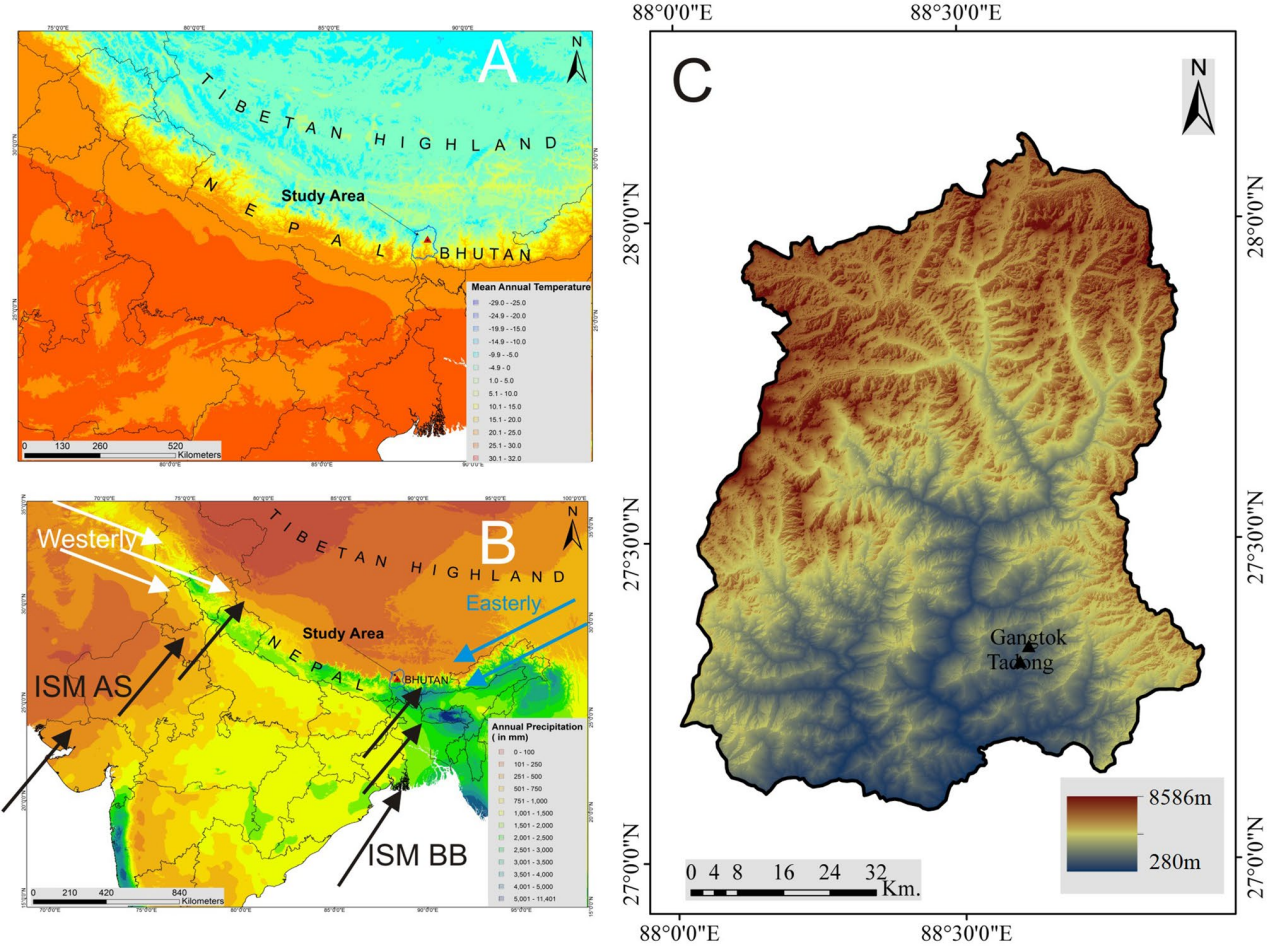
The relative location of the study area. **(A)** Mean annual temperature^22^, **(B)** annual precipitation^22^ and **(C)** the Digital elevation Model (SRTM) of the Sikkim Himalaya along with the location of Gangtok and Tadong observatories. ISM (Indian Summer Monsoon) AS denotes to Arabian Sea branch and ISM BB to Bay of Bengal branch. The figure is prepared using ArcGIS 10.2.

The study area lies within the lower Teesta River basin in Sikkim Himalaya, India (Fig. 1). The basin shares its boundaries with Bhutan by the Chola range in the East, with Nepal by Singalila range in the west, and Tibet by Greater Himalaya and Chola range on the north and north-east. The famous Kanchenjunga peak (8586 m) shares the western boundary of the study area. The altitude varies between ~ 280 and 8,586 m from south to north transects. The spectacular relief of the study area controls the atmospheric processes in the region. There are only two weather observatories found in the study area, namely; Gangtok and Tadong which are located at 27^o^20′ N, 88^o^37′ E and 27^o^20′ N, 88^o^38′ E, respectively, for long term records. The stations are situated on a ridge in the Teesta valley confined by the tributaries; *Roro Chu* and *Ranikhola*. Gangtok observatory is located in the city area of Gangtok, the capital city of the state of Sikkim and is located at an elevation of ~ 1812 m whereas Tadong is situated at ~1322 m. The climate varies with the altitudinal zones. Precipitation is mainly concentrated during the summer and monsoon period, lasting from May to October. Higher temperatures are recorded from June to August, while the lower ones prevail from December to January which would also witness snowfall. The area is a transitional zone between the Arabian Sea branch and Bay of Bengal branch of the Indian Summer Monsoon (Fig. 1B). During monsoon, the area faces the thrust of both the branches resulting in heavy orographic rainfall in the region. The elevated topography of the Higher Himalayas further controls the distribution of rain by the aspect and height of the valleys.

The upper catchment of the Teesta basin is dominated by the glacier processes and occupies an area of ~ 346.63 km^2^. The basin has more than 100 glaciers of varying size, of which Zemu (26 km) is the largest one^21^. Many glaciers including *Zemu* and *E*. *Rathong* originate from the eastern slopes of Kanchenjunga and enter into the study area feeding the two major river systems of the region i.e. Teesta and Rangit.

## Material and methods

### Datasets

The study used monthly and annual temperatures (minimum, maximum and average) and precipitation data to analyze climatic trends for Gangtok and Tadong stations. The climatic data of temperature and precipitation were procured from the Indian Meteorological Department (IMD), the most accepted and reliable source of climatic data in the Indian scenario. As a standard practice, the IMD carries out occasional quality checks to make the data error-free to ensure the quality before releasing it in the public domain^23^. The duration of these data lasts 57 years (1961–2017) for Gangtok whereas for Tadong it is relatively limited, 30 years (1981 to 2010). Any missing value, which was lesser than 1% in the data series has been filled up by using the temporal interpolation method as suggested by Bhutiyani et al. (2007)^14^. Pettitt's test is used to inspect the homogeneity of the data sets for both the stations and it reveals that the data series of Gangtok station is inhomogeneous and has a point of change while the data series for Tadong is homogenous. The discrepancy in terms of homogeneity of the data for both the stations may be attributed to their relative locations. Gangtok is an urban area that may have affected homogeneity by human development processes e.g. urban heat island, whereas Tadong is located a few kilometers outside Gangtok city.

### Trend analysis

The study used the Mann–Kendall non-parametric test to know the presence or absence of a trend in climatic parameters along with Sen’s slope estimator to calculate the magnitude of shift over time. These methods are widely used in the trend analysis of climatic variables^16,18,23–29^. The study used the Mann–Kendall test as it overcomes the abrupt breaks caused by inhomogeneity in time series and does not require the data to be normally distributed^30^. The test validates the null hypothesis, H_0_ (no trend) against the alternative hypothesis H_1_, assuming that there is a positive or negative trend^31^. It is calculated as follows after Mann–Kendall.

1 $$S=\sum_{k=1}^{n-1} \sum_{i=k+1}^{n}sign({y}_{i}-{y}_{k}),$$

where,

2 $$sign\left(x\right)=\left\{\begin{array}{ll}-1, &\quad if\,\, x<0,\\ 0,&\quad if\,\, x=0,\\ 1,&\quad if\,\, x>0.\end{array}\right.$$

The null hypothesis, H_0_ will be rejected if |S| is less than the specified significance level of the test, α in the table of probability^29^. For a two-tailed test, the tabled probability level corresponding to the absolute value of S is doubled before the comparison with significance level α^32^. This method is applicable if the number of observations in the time series data is 40 or less, i.e., *n* ≤ 40. For *n* larger than 40, the normal approximation test is used^29^. First, we compute the variance of S as follows.

3 $$Var\left(S\right)=\frac{1}{18}[n\left(n-1\right)\left(2n+5\right)-\sum_{p=1}^{g}{t}_{p}\left({t}_{p}-1\right)\left(2{t}_{p}+5\right)],$$

where $\mathrm{g}$ represents the number of tied groups and t_p_ is the number of observations in the p^th^ group. For example, in the sequence of measurements in time {21, 18, 23, 18, 24, 30, 6, 30, 24, 24, 30, 23, 29, 30} we have g = 4 tied groups, for which t_1_ = 2 for the tied value 18, t_2_ = 2 for the tied value 23, t_3_ = 3 for the tied value 24, and t_4_ = 4 for the tied value 30. When there are ties in the data due to equal values or non-detects, it is adjusted by a tie correction method^33^. The MK test statistic (Z_MK_) is computed as follows^34^.

4 $${Z}_{MK}=\left\{\begin{array}{ll} \frac{S-1}{ \sqrt{VAR(S)}}&\quad if\,\, S>0,\\ 0&\quad if\,\,S=0, \\ \frac{S+1}{\sqrt{VAR\left(S\right)}} &\quad if\,\, S<0.\end{array}\right.$$

The probability (p-value) associated with this normalized test statistic is computed using probability density function for a normal distribution. The value of mean and standard deviation is 0 and 1 respectively and is given by:

5 $$f\left(z\right)=\frac{1}{\sqrt{2\pi }}{e}^{-\frac{{z}^{2}}{2}}$$

Kendall's Tau ($\tau$): Kendall's tau is an important measure with the Mann–Kendall test to measure the correlation between two ranked variables and can be defined as follows^35^.

6 $$\tau =\frac{C-D}{C+D} = \frac{C-D}{\frac{n(n-1)}{2}}.$$

where C and D represent the number of Concordant and Discordant pairs, respectively. A pair of the point is concordant if the rank of the second variable is greater than the rank of the first variable and discordant if the rank of the second variable is equal or less than the rank of the first variable^35^.

All the tests are carried out by using a 95% confidence level. The testing of the null hypothesis is based on a comparison of the P-value (two-tailed) and alpha value. If the exact p-value could not be computed then an approximation has been used to compute the p-value.

### Sen’s slope estimator

Sen’s slope estimator was proposed by Sen^36^. This slope may also be estimated by computing the least-square estimate using the slope of the linear regression method. The result of the linear regression method is highly sensitive to gross error in the data and outliers present in the data. However, Sen’s slope estimator is less affected by outliers and robust in case of missing data. Let (*t*_1,_ *x*_1_), (*t*_2,_ *x*_2_), …… (*t*_*n*_*, x*_*n*_) are the *n* observation of time series data where *x*_*i*_ is observed at time *t*_*i*_. Now calculate the slope *M*_*i*_,_*j*_ for all pair of point (*x*_*j*_ , *x*_*i*_), *i* < *j* using formula as^37^.

7 $${M}_{i,j}=\frac{{x}_{j}-{x}_{i}}{{t}_{j}-{t}_{i}}$$

For *n* number of observations, there is *N* = *n* (*n*-1)/2 number of slopes. Arrange these *N* slopes in ascending order and let it be *s*_1_, *s*_2_, …… *s*_*N*_. The median of slopes is given by:

8 $$Median=\left\{\begin{array}{c}{\left(\frac{N+1}{2}\right)}^{th }term, \quad when\,N\,is\,Odd,\\ \frac{{\left(\frac{n}{2}\right)}^{th} term+{\left(\frac{n}{2}+ 1\right)}^{th}term}{2}, \quad when\,N \,s \, even.\end{array}\right.$$

This calculated median is the slope estimate and is known as Sen’s slope estimator.

### Normal distribution curve

The normal distribution is helpful to explain a family of continuous probability distributions. Each distribution has the same general shape but differing in their location (mean) and scale parameters (standard deviation)^38^. The graph of its probability density function is the symmetric and bell-shaped curve^39^. The normal distribution is the most established model to characterize the quantitative variation of the original data. The normal distribution is also known as the Gaussian distribution since it was first discovered by the German mathematician, Gauss (1777–1855). A continuous random variable *X* is said to have normal distribution if its probability distribution function is as follows^38,39^.

9 $$f\left(x\right)=\frac{1}{\sigma \sqrt{2\pi }}{e}^{-{(x-\mu )}^{2}/2{\sigma }^{2}}$$

where µ is the mean and σ is the standard deviation of the distribution respectively. The normal distribution is an appropriate method to analyze the pattern of lower, upper, and extreme events in terms of temperature and precipitation. It completely dependents on mean and variance and hence any change in the shape of its curve will reflect the changes in the distribution of events^40^. Additionally, the One Way ANOVA test is used at a 95% confidence level to check whether there are any statistically significant changes in mean for given data trends, which may suggest the shift in the means of minimum, maximum, average temperatures, and precipitation.

### Cumulative sum (CUSUM) control chart

The CUSUM (CUmulative SUM) control chart is a sequential analysis technique used for change detection^41^. It exhibits the cumulative sums of the deviations from a target value. The CUSUM would result in zero if there is no deviation from the target value in the data series. The more deviation from the target value reflects the more unevenness of variables^13,42^. Several studies have used the Cusum chart to detect the point of change in climatic data series^13,42^.

Let *x*_1_, *x*_2_… *x*_*n*_, be the n observations observed on some time scale. Let τ be the target mean of the observations. The cumulative sum for each observation is calculated as follows^43^.

$${C}_{0}= 0$$

10 $${C}_{i}= {C}_{i-1}+\left({x}_{i}-\tau \right), \quad i=1, 2 \dots .. n$$

An expert can select any value as a target mean using his/her expertise. The mean of the observations can also be used as the target mean. After finding all *c*_*i*_, plot the *c*_*i*_ and the resultant chart is a CUSUM chart.

The tabular CUSUM chart is an algorithmically approach for change detection. For a target value τ and a reference value *k*, define the following two measures:

11 $${C}_{i}^{+}=\mathrm{max}\{0,{x}_{i}-\left(\tau +k\right)+{C}_{i-1}^{+}\}$$

12 $${C}_{i}^{-}=\mathrm{max}\{0, \left(\tau -k\right)-{x}_{i}+{C}_{i-1}^{-}\}$$

Starting with $${C}_{0}^{+}={C}_{0}^{-}=0$$.

If the ${C}_{0}^{+} or \,{C}_{0}^{-}$ exceeds a decision interval H then the process is out of control. To detect a change of process mean to a new mean say τ_1_, then the value of *k* is calculated using the following:

13 $$k=\frac{|{\tau }_{1}-\tau |}{2}$$

In the present study, the target value is the long period average of each data series. Deviation from the target value is compared with the upper and lower limits of Cusum that is 3σ for this study.

## Results

### Trend analysis of climatic parameters at Gangtok station

**Figure 2:**
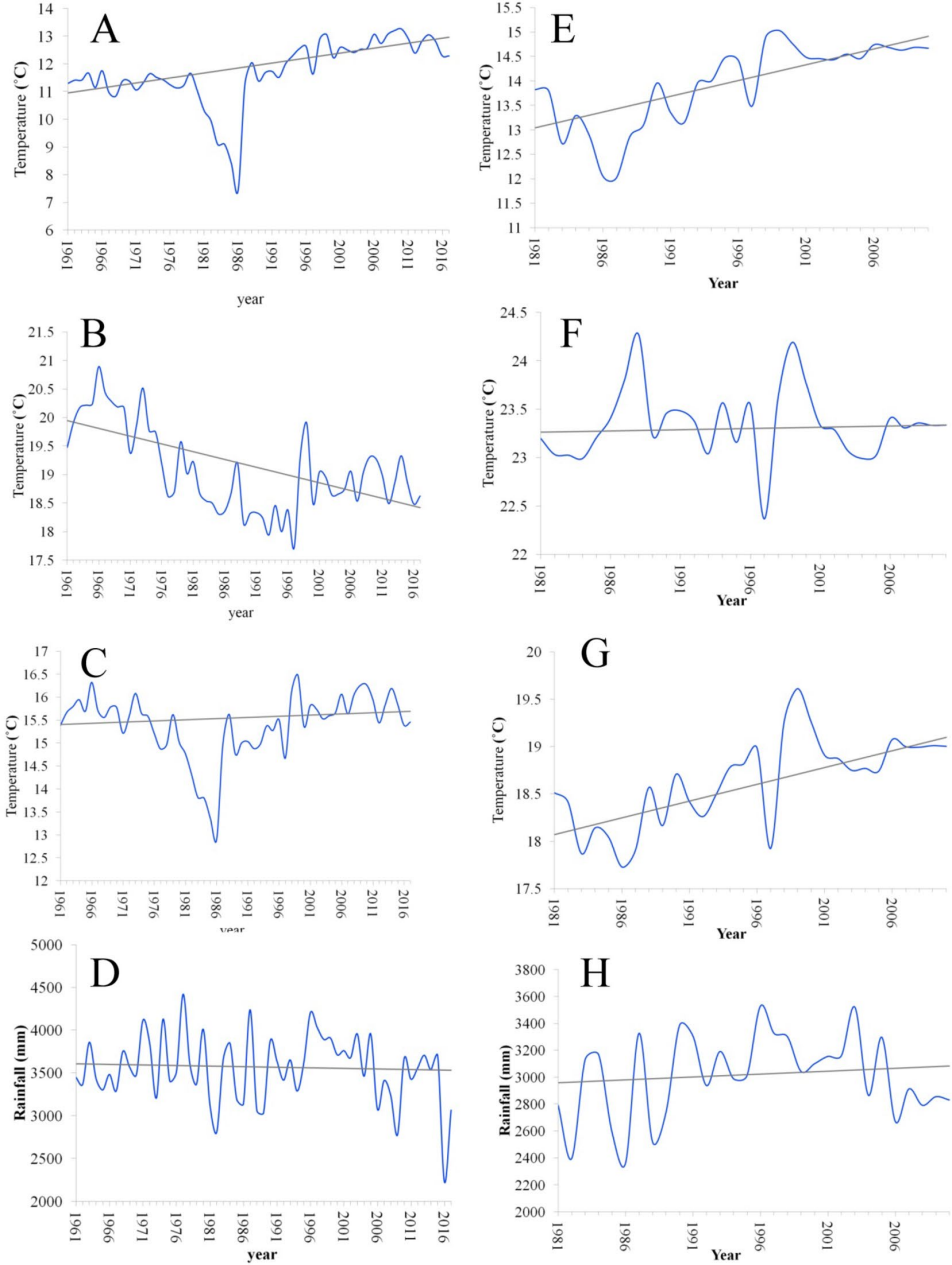
Trends of **(A)** minimum temperature, **(B)** maximum temperature, **(C)** average temperature, **(D)** precipitation at Gangtok station, and **(E)** minimum temperature, **(F)** maximum temperature, **(G)** average temperature, **(H)** precipitation at Tadong station.

Table 1 represents the trends in temperatures and precipitation at Gangtok and Tadong stations. The study recorded a positive trend in the minimum temperature at Gangtok as the computed p-value (< 0.0001) of the Mann–Kendall test is lower than the significance level alpha (0.05). The p-value suggests that the null hypothesis H0 is rejected (no trend), and the alternative hypothesis H1 is accepted (a trend is recorded). The Sen’s slope estimator furthers support the finding and suggests an increase of 2.05 ˚C at the rate of 0.036 ˚C year^−1^ from 1961 to 2017 (Table 1 and Fig. 2A). The minimum temperature recorded a mean of 11.69 ˚C along with a standard deviation (SD) and coefficient of variation (CV) of 1.18 ˚C and 10.09%, respectively for the observed period.

**Table 1:** Trends of annual temperatures (˚C) and precipitation (mm) at Gangtok (1961–2017) and Tadong (1981–2010) using the Mann–Kendall trend test and Sen’s slope estimator.

	Min.	Max.	Mean	Std. deviation	CV (%)	Kendall's tau	S	Var(S)	Z_MK_	p-value (two-tailed)	alpha	Trend	Sen's slope
**Gangtok**													
Min. temp	7.43	13.27	11.69	1.18	10.09	0.51	807.00	21099.67	5.55	< 0.0001	0.05	Y +	0.036
Max. temp	17.72	20.89	19.09	0.75	3.92	− 0.36	− 568.00	21100.67	− 3.90	< 0.0001	0.05	Y−	− 0.027
Avg. temp	12.90	16.48	15.39	0.71	4.61	0.10	166.00	21102.67	1.14	0.26	0.05	N	0.005
Precipitation^a^	2229.40	4422.50	3547.00	398.84	11.24	− 0.03	− 54.00	21102.67	− 0.36	0.72	0.05	N	− 1.354
**Tadong**													
Min. temp	12.02	15.03	13.93	0.85	6.10	0.59	255.00	3141.67	4.53	< 0.0001	0.05	Y +	0.065
Max. temp	22.37	24.28	23.34	0.37	1.58	0.05	22.00	3140.67	0.37	0.71	0.05	N	0.002
Avg. temp	17.73	19.61	18.63	0.47	2.52	0.53	230.00	3140.67	4.09	< 0.0001	0.05	Y +	0.035
Precipitation^a^	2361.30	3529.50	3006.54	313.36	10.42	0.05	21.00	3141.67	0.36	0.72	0.05	N	4.358

Y− refers to negative and Y + indicates positive and N indicates no trend in the data.

^a^Annual precipitation at the observed stations.

Differing from the trend of minimum temperature, the maximum temperature showed a negative trend as the computed p-value (< 0.0001) of the Mann–Kendall test is lower than the significance level alpha (0.05). Similar to the minimum temperature, in case of the maximum temperature, the null hypothesis H0 is rejected and the alternative hypothesis H1 is accepted, but with a negative trend. The Sen’s slope estimator indicates a total decline of − 1.53 ˚C at the rate of − 0.027 ˚C year−^1^ which is contrary and smaller than the minimum temperature for the same assessment period (Table 1 and Fig. 2B). The maximum temperature recorded a mean of 19.09 ˚C along with a standard deviation of 0.75 ˚C. The maximum temperature showed lesser variation as CV stands at 3.92% in comparison to the minimum temperature for the same assessment period.

The average annual temperature showed no trend with a mean of 15.39 ˚C for the same assessment period. It registered an SD of 0.71 ˚C and a CV of 4.61% suggesting lesser variability (Table 1 and Fig. 2C). Similarly, the annual precipitation also did not indicate any trend. The mean for the annual precipitation was recorded 3,547 mm along with SD of 398.84 mm for the assessed period at Gangtok (Fig. 2D). The recorded CV for precipitation was 11.24% indicating a larger variability in the precipitation at Gangtok.

In brief, Gangtok recorded a positive trend for the minimum temperature, a negative trend for the maximum temperature, and reflected no trend in the average temperature and precipitation. The minimum temperature and precipitation exhibit larger variability.

### Trend analysis of climatic parameters at Tadong station

Analysis of the minimum temperature at Tadong station suggests a positive trend (p-value < 0.0001) along with a mean of 13.93 ˚C (greater than the mean of minimum temperature at Gangtok) for the observed period from 1981 to 2010. However, it recorded lesser variation as SD and CV stand at 0.85 ˚C and 6.10%, respectively. The trend is very much evident in Fig. 2E, which shows a sharp rise in the minimum temperature. The Sen’s slope estimator further supports the finding as it shows an increase of 1.95 ˚C with a rate of 0.065 ˚C year^−1^ for the observed period. The rise in minimum temperature at Tadong is similar to the rise in minimum temperatures trend at Gangtok.

Unlike the negative trend at Gangtok, Tadong recorded no trend in maximum temperature (Table 1, Fig. 2F). Comparatively, Tadong recorded a higher mean of 23.34 ˚C in maximum temperature with the least variation as SD and CV stand at 0.37 ˚C and 1.58%, respectively for the observed period.

The aforementioned divergent trend continues between Gangtok and Tadong in the case of the average temperature as well. As the Mann–Kendall test suggests a positive trend (p-value of < 0.0001) in average temperature at Tadong but no trend for Gangtok. The Sen’s slope estimator showed an increase of 1.05 ˚C with a rate of 0.035 ˚C year^−1^ for the same assessment period (Fig. 2G). The mean of average temperature at Tadong is found to be 18.63 ˚C (higher than the mean of average temperature at Gangtok) along with a standard deviation and CV of 0.47 ˚C and 2.52%, respectively.

The annual precipitation at Tadong was recorded at 3,006.54 mm (lower than Gangtok) and fluctuated with a standard deviation of 313.36 for the assessment period. Mann–Kendall test indicates no trend in the precipitation at Tadong as well (Fig. 2H). The estimated CV value (10.42%) suggests a similar variation in precipitation as recorded in the case of Gangtok too.

### Pattern of extreme event distribution

**Figure 3:**
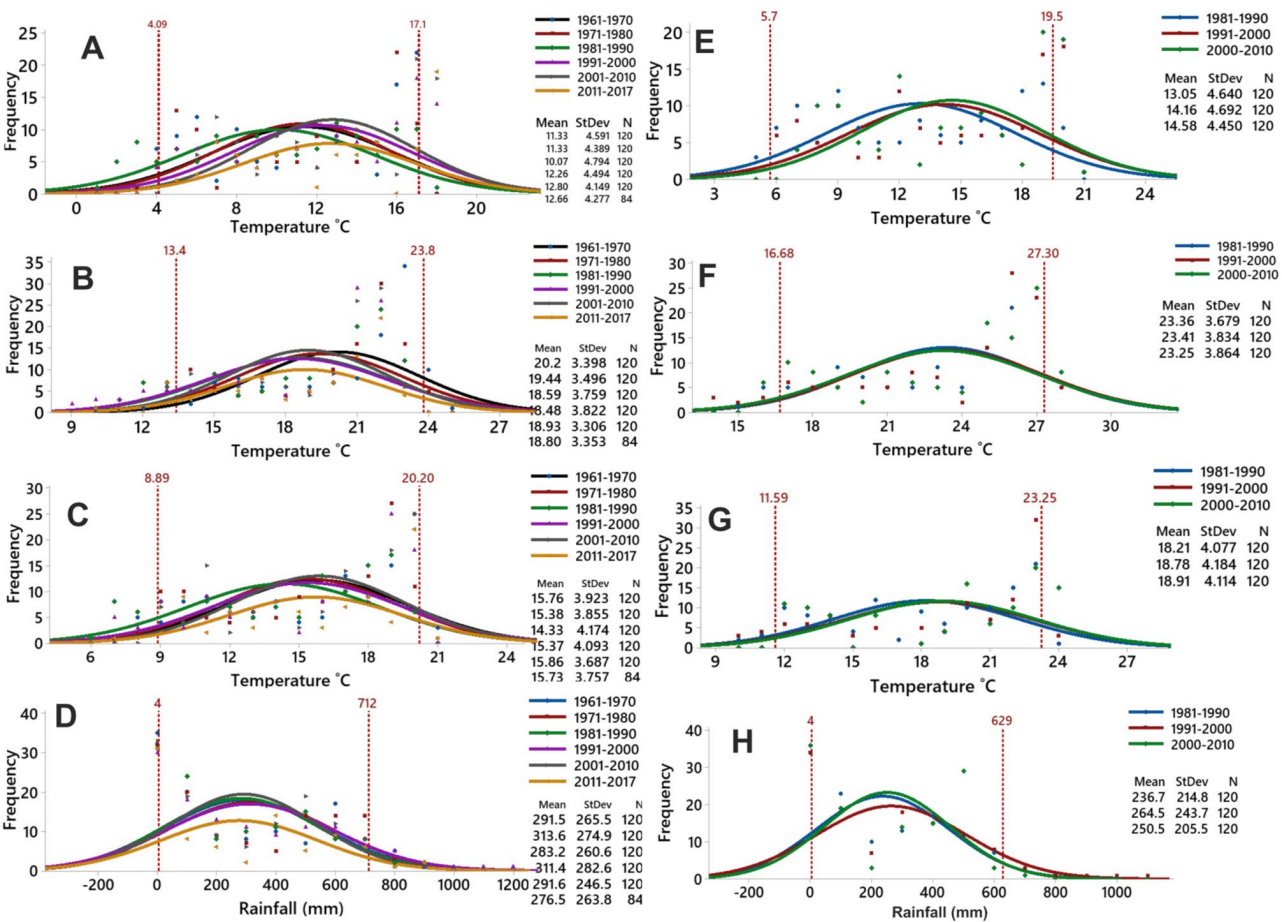
The pattern of distribution of events of **(A)** minimum temperature, **(B)** maximum temperature, **(C)** average temperature, and **(D)** precipitation at Gangtok, and **(E)** minimum temperature, **(F)** maximum temperature, **(G)** average temperature, and **(H)** precipitation at Tadong station on decadal scale.

The study analyzed the frequency of upper-end and lower-end events of monthly temperatures and precipitation on decadal scale at Gangtok and Tadong stations. Figure 3 shows the normal distribution fit for the minimum, maximum and average temperatures along with precipitation. The two vertical red lines at the 5th percentile and the 95th percentile demarcate the lower end and upper-end extreme events (Fig. 3). In the distribution curves, the decades of 1961–1970 for Gangtok and 1981–1990 for Tadong are taken as the base decade (termed as the first decade) to assess the direction of shift of extreme events. The One Way ANOVA test suggests a statistically significant shift in the means of minimum, maximum, and average temperatures over the decades at Gangtok as the computed p-values of these shifts are < 0.0001 for minimum temperature, 0.002 for maximum temperature and 0.033 for average temperature, lower than the significance level alpha value (0.05). In the case of Tadong, only minimum and average temperatures showed a significant shift with p-values of 0.030 and 0.037, respectively. However, any statistically significant shift was not detected in the case of precipitation at both the stations. The decadal analysis of the monthly minimum temperature at Gangtok suggests that the means of the second (1971–1980) and third (1981–1990) decades have shifted continuously to the left (toward the lower-end) of the mean of the base decade (1961–1970) which indicate that the probability of lower-end events has increased during these two decades. However, during the fourth (1991–2000) and fifth (2001–2010) decade, the means have shifted continuously towards the right of the base decade denoting an increased probability of upper-end events (Fig. 3A) at Gangtok. Similar types of shifts have been detected at the Tadong station for the decades of 1991–2000 and 2001–2010 which also indicate the increased probability of upper-end events (Fig. 3E). Differing from the pattern of the minimum temperature, the means of the maximum temperature of all the decades have shifted to the left of the mean of the base decade at Gangtok, indicating an increased probability of lower-end events (Fig. 3B). The same pattern of the shift towards the lower end is observed in the maximum temperature at Tadong as well but with apparently lower magnitude (Fig. 3F).

The average temperature at Gangtok, during the second (1971–1980) and third (1981–1990) decade, has shifted to the left of the base decade (1961–1970), indicating an increased probability of lower-end events. However, from the fourth (1991–2000) decade onwards the means and curve of the average temperature started to shift towards the right of the preceding decades whereas in the decade of 2001–2010 the shift turns to the right of the base decade (Fig. 3C). In the case of Tadong, the means and curve of the average temperature have shifted towards the right of the base decade during 1991–2000 and 2001–2010 which is similar to Gangtok station (Fig. 3G). In the case of precipitation, no pattern was noticed (Fig. 3D, H). To summarize, it is evident from Fig. 3 that the second and third decade witnessed an increase in the lower-end events at Gangtok whereas the upper-end event dominated from the fourth decade onward at Gangtok as well as at Tadong.

### Detection of variability and change in the climatic parameters

**Figure 4:**
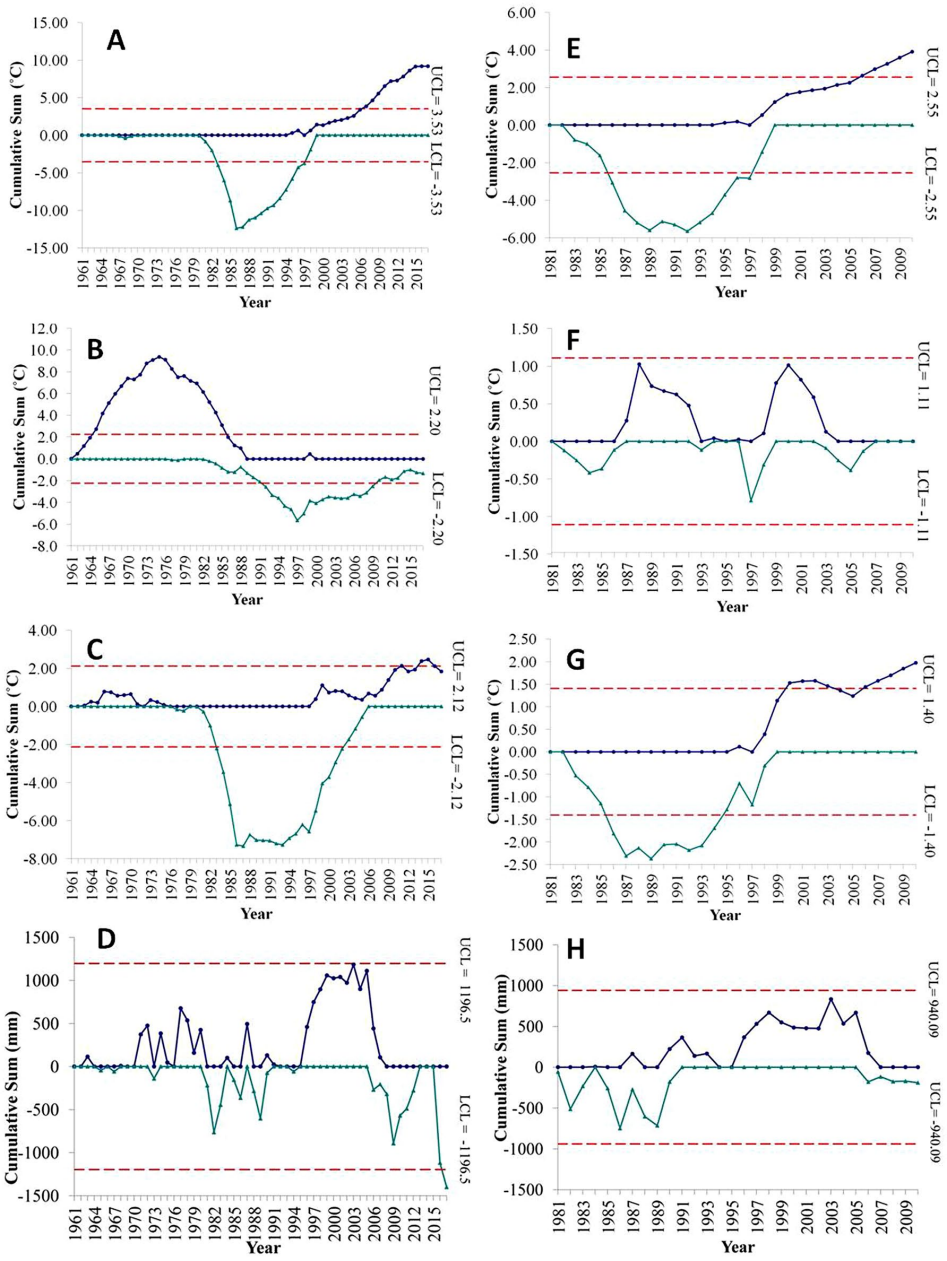
CUSUM graphs show the point of change at Gangtok station—**(A)** minimum temperature, **(B)** maximum temperature, **(C)** average temperature, **(D)** precipitation and at Tadong station—**(E)** minimum temperature, **(F)** maximum temperature, **(G)** average temperature, **(H)** precipitation.

The representative charts (CUSUM) in Fig. 4 show the cumulative sums of the deviation of the observed minimum, maximum, average temperatures and precipitation at both the stations. Figure 4A, E shows that the minimum temperature dipped down the lower threshold of the Cusum chart and hence suggests that the minimum temperature at both Gangtok and Tadong recorded a phase of cooling for almost a similar period from 1983 to 1995. However, it is noticed that the minimum temperature started to shoot up after 1995 and crossed the upper cusum limit by 2005 at both the stations (Fig. 4A, E). Other than these phases of warming and cooling, the minimum temperature was within the threshold limit, hence no trend was observed.

Interestingly, the maximum temperature shows a warming period from 1965 to 1984 when the minimum temperature showed no trend at Gangtok. After the warming phase in maximum temperature, Gangtok recorded a continuous cooling phase from 1991 to 2008. However, no particular phase was detected in case of maximum temperatures at Tadong station as the temperature fluctuated within the upper and lower limits of the cusum (Fig. 4B, F), with more positive deviation from the normal being observed in Fig. 4F.

The average temperature suggests a cooling phase from 1983 to 2000 at Gangtok station. A similar cooling phase in average temperature is also detected at Tadong station from 1986 to 1995. However, a warming phase started after 2005 at bothTadong and Gangtok stations (Fig. 4C, G). Precipitation values did not cross the limits of the upper and lower Cusum chart at both the station (Fig. 4D, H). In brief, the warming is clearly visible in the case of the minimum and average temperatures.

## Discussion

The present study discusses the pattern of climatic trends, monthly extreme events and climatic variability in the study area. The study records similar positive trends (2.05 ˚C & 1.95 ˚C) in the minimum temperatures at both Gangtok and Tadong observatories. In the case of maximum temperature, Gangtok recorded a negative trend (− 1.53 ˚C) while Tadong showed no trend. The average temperature showed no trend at Gangtok and a positive trend (1.05 ˚C) at Tadong for the respective assessment periods. The dissimilarity in the case of maximum and average temperatures between both the stations may be attributed to the different duration of analyzed data for each station. However, it is evident from the analysis of data of overlapping periods for both the stations, that similarities do exist in case of maximum and average temperatures as well for both the stations (Supplementary table S1). During the overlapping periods, the maximum temperatures and average temperatures at Gangtok show no trend and a positive trend, respectively, similar to the trends at Tadong station. The higher increasing trend of minimum temperatures and lower or no trend of maximum temperature in the Sikkim Himalaya is also fully or partially supported by the results of other studies on the northeast region of India as an increase of ~ 2.1 ˚C^45^ in the minimum temperature and lower or not-systematic changes in maximum temperature have been reported^44–46^. Precipitation did not record any particular trend at both the stations.

To validate the traits of these trends and variations, the present study analyzed the pattern of monthly events of the climatic parameters at both the stations. In the case of the minimum temperatures, the probability of extreme events on the upper-end has increased continuously from the base decades at both the stations which may explain the observed positive trend. However, the maximum temperature recorded the dominance of the lower-end events for the initials decades but showed a gradual shift towards the upper-end in the later decades at both Gangtok and Tadong stations. The average temperature recorded dominance of lower-end events in the early 1980s to 1990s at Gangtok; however, it also recorded a shift towards the upper-end events thereafter at Gangtok as well as at Tadong. The observed warming trends in the later decades (Fig. 3) at both the stations may be attributed to the shift in the monthly events towards the upper-end.

The positive trends in the minimum temperatures and negative or no trend in the maximum temperatures at both the stations can be understood with the help of seasonal characteristics of the Indian Summer Monsoon (ISM) and western disturbances in this part of the Himalaya. Eastern Himalaya (present study area) experiences dominance of Bay of Bengal branch of ISM, which remains active for a longer period from June to October (4–5 months), resulting in extensive cloud cover and heavy downpours^47^. Possibly, it results in a controlled maximum temperature during the peak summer season in the eastern Himalayas. However, during the winter season, the negligible impact of the western disturbances is observed in the Eastern Himalaya and the sky remains comparatively clear, which might have resulted in the increased minimum temperatures as the same is also reported in a similar study on the Sikkim region^18^.

A comparative analysis with existing studies on other parts of the Himalaya including Nepal and Bhutan has been helpful to develop a climatic synthesis for the whole Himalayan region. The climatic studies on Nepal and central Himalaya conducted by Shrestha et al. (1999) and Kattel and Yao (2013)^12,13^, Bhutiyani et al. (2007) on north-west Himalaya^14^ and Shekhar et al. (2010) on the western Himalaya suggested warming trends at diverse rate^48^. However, the western Himalayas and the central Himalayas observed contrary trends in minimum and maximum temperatures in comparison to the eastern Himalayas. Bhutiyani et al. (2007) suggested a positive trend in both the maximum (1.6 ˚C) as well as minimum (1.1 ˚C) temperatures, with the maximum temperature increasing more rapidly in northwestern Himalaya^14^. Similarly, in the case of central Himalaya too, Kattel and Yao (2013) suggested a higher magnitude of warming in the maximum temperatures (1.95 ˚C) and exhibit larger variability such as positive, negative, or no change in the case of minimum temperatures. Whereas, on account of the positive trend in maximum temperature in western and central Himalaya, the present study differs and suggests negative and no trends in the eastern Himalaya. Furthermore, the present study observed a positive trend in the minimum temperatures in the eastern Himalaya which is contrary to the central and western Himalaya. This antinomy in the trends of maximum and minimum temperatures may be assumed to originate from the different precipitation regimes dominating over the western and eastern Himalayas. Such results suggest the control of ISM, western disturbances and resultant varying duration of cloud cover across the Himalayas^47^. The shorter duration of ISM in central and western Himalaya provides clear sky in the summer season giving enough time to heat up the landscape attaining the peaks in the temperatures which may in turn result in the increased maximum temperatures in the region. Additionally, the contrary case of the decreasing minimum temperatures in the western and central Himalaya could be attributed to the comparatively increased cloud cover during the winter season due to western disturbances in the region.

On account of precipitation, the study suggests no particular trend which is in conformity with the works of Borgaonkar and Pant (2001) that detects no precipitation trend over the western Himalayas^49^. Shrestha et al. (2000) also suggested similar results from Nepal Himalayas^15^. However, large ambiguity exists on part of the understanding of the precipitation trend in the Himalayas. As Kumar et al. (2005) on one hand suggests a positive trend in the annual rainfall and on the other, report a negative trend in monsoonal rainfall in the parts of the Himachal Himalayas^50^. Basistha et al. (2009) reported temporal variation and suggested increasing trend up to 1964 (corroborating with all India and nearby plains), followed by a decreasing trend from 1965 to 1980 in parts of Uttrakhand Himalaya^16^. On the other hand, Bhutiyani et al. (2010) monitoring seasonal variation indicated insignificant increasing trend in the winter precipitation and a decreasing trend in the monsoon and overall annual precipitation from 1866 to 2006 in the northwestern Himalaya^17^.

The reported warming has left its imprints on the water resources available in the form of glaciers in the region. Several studies have reported on the glaciers of this region and found that glaciers have lost ~ 20% area on an average in the recent decades^51–53^. Similarly, studies have also reported an expansion up to ~ 24% in the size of glacial lakes which pose a great threat to the down valley in the form of glacial lakes outburst floods^54,55^. Moreover, the reported warming may cause shifts in the altitude based climatic zones in the region that may affect ecological settings and agricultural practices, directly or indirectly. The impacts of warming and the strategies to tackle the same are very well evident in the adopted climate change action plans by the Sikkim state^56^.

## Conclusions

Overall, the study suggests that the minimum temperatures recorded a positive trend at both the stations. However, the maximum temperatures have shown a negative trend for the entire assessed period and no trend for the overlapping period at Gangtok. The maximum temperatures have recorded no trend at Tadong as well. The average temperatures have recorded no trend for the entire assessed period but a positive trend for the overlapping period at Gangtok and similar positive trends at Tadong. The warming conditions were more prominent in the last decades, better reflected in the minimum temperature. These conditions are contrary to the trend in the western and central Himalayan regions which further suggests different climatic drivers in these regions. Precipitation remains fluctuating throughout the observed period.

## Supplementary information

Supplementary information.

## References

[1] Jones PD, Wigley TML, Wright PB (1986). Global temperature variations between 1861 and 1984. Nature.

[2] Jones PD (1986). Northern hemisphere surface air temperature variations: 1851–1984. J. Clim. Appl. Meteorol..

[3] Hansen J, Lebedeff S (1987). Global trends of measured surface air temperature. J. Geophys. Res..

[4] Hansen J, Lebedeff S (1988). Global surface air temperatures: Update through 1987. Geophys. Res. Lett..

[5] Diaz HF, Bradley RS, Eischeid JK (1989). Precipitation fluctuations over global land areas since the late 1800’s. J. Geophys. Res. Atmos..

[6] Dore MMHIM (2005). Climate change and changes in global precipitation patterns: what do we know?. Environ. Int..

[7] Bates, B. C., Kundxewicz, Z. W., Wu, S. & Palutikof, J. P. Observed and projected changes in climate as they relate to water. *IPPC Tech. Pap. 4. Clim. Chang. Water* 20 (2008).

[8] Easterling, W.E., Aggarwal, P.K., Batima, P., Brander, K.M., Erda, L., Howden, S.M., Kirilenko, A., Morton, J., Soussana, J.-F., Schmidhuber, J., Tubiello, F. N. Food, fibre and forest products. *Climate Change 2007 Impacts, Adaptation Vulnerability Contribution Working Group II to Fourth Assessment Report Intergovernment Panel Climate Change* 273–313 (2007).

[9] Diaz HF, Grosjean M, Graumlich L (2003). Climate variability and change in high elevation regions: Past, present and future. Clim. Change.

[10] Dash SK, Jenamani RK, Kalsi SR, Panda SK (2007). Some evidence of climate change in twentieth-century India. Clim. Change.

[11] Sivakumar, M. V. K. & Stefanski, R. Climate change in South Asia. in *Climate Change and Food Security in South Asia* (ed. Lal, R; Sivakumar, M.V.K.; Faiz, M.A., Mustafizur Rahman, A.H.M., Islam, K. R.) 13–30 (Springer, 2011). 10.1007/978-90-481-9516-9_2

[12] Shrestha AB, Wake CP, Mayewski PA, Dibb JE (1999). Maximum temperature trends in the Himalaya and its vicinity: An analysis based on temperature records from Nepal for the period 1971–94. J. Clim..

[13] Kattel DB, Yao T (2013). Recent temperature trends at mountain stations on the southern slope of the central Himalayas. J. Earth Syst. Sci..

[14] Bhutiyani, M. R., Kale, V. S. & Pawar, N. J. Long-term trends in maximum , minimum and mean annual air temperatures across the Northwestern Himalaya during the twentieth century. **85**, 159–177 (2007).

[15] Shrestha AB, Wake CP, Dibb JE, Mayewski PA (2000). Precipitation fluctuations in the Nepal Himalaya and its vicinity and relationship with some large scale climatological parameters. Int. J. Climatol..

[16] Basistha A, Arya DS, Goel NK (2009). Analysis of historical changes in rainfall in the Indian Himalayas. Int. J. Climatol..

[17] Bhutiyani MR, Kale VS, Pawar NJ (2010). Climate change and the precipitation variations in the northwestern Himalaya: 1866–2006. Int. J. Climatol..

[18] Sharma, R. K. & Shrestha, D. G. Climate perceptions of local communities validated through scientific signals in Sikkim Himalaya, India. *Environ. Monit. Assess.* **188** (2016). 10.1007/s10661-016-5582-y 27650439

[19] Rahman, H., Karuppaiyan, R., Senapati, P. C., Ngachan, S. V & Kumar, A. Mid-hills of Sikkim and strategies for mitigating possible. in *Climate Change in Sikkim - Patterns, Impacts and Initiatives* (ed. M. L. Arrawatia, S. Tambe) 19–48 (Information and Public Relations Department, Government of Sikkim, 2008).

[20] Seetharam, K. Climate change synthetic scenario over Gangtok. in *Climate Change in Sikkim - Patterns, Impacts and Initiatives* (ed. M. L. Arrawatia, S. Tambe) 1–18 (Information and Public Relations Department, Government of Sikkim, 2008).

[21] Sharma MC, Deswal S, Kumar P (2009). Himalayan glacier and climate change: a case of misunderstood science. Think India Q..

[22] Hijmans RJ, Cameron SE, Parra JL, Jones PG, Jarvis A (2005). Very high resolution interpolated climate surfaces for global land areas. Int. J. Climatol..

[23] Kumar V, Jain SK, Singh Y (2010). Analysis of long-term rainfall trends in India. Hydrol. Sci. J..

[24] Jain SK, Kumar V (2012). Trend analysis of rainfall and temperature data for India. Curr. Sci..

[25] Lacombe, G. & Mccartney, M. Uncovering consistencies in Indian rainfall trends observed over the last half century. 287–299 (2014). 10.1007/s10584-013-1036-5

[26] Roy AD (2015). Temperature and rainfall dynamics in Penganga sub watershed, Maharashtra. Indian J. Spat. Sci..

[27] Atta-ur-Rahman & Dawood, M. Spatio-statistical analysis of temperature fluctuation using Mann–Kendall and Sen’s slope approach. *Clim. Dyn.* (2016). 10.1007/s00382-016-3110-y

[28] Nkiaka E, Nawaz NR, Lovett JC (2016). Analysis of rainfall variability in the Logone catchment, Lake Chad basin. Int. J. Climatol..

[29] Gilbert, R. O. *Statistical Methods for Environmental Pollution Monitoring*. (Wiley, 1987).

[30] Tabari H, Marofi S, Aeini A, Talaee PH, Mohammadi K (2011). Trend analysis of reference evapotranspiration in the western half of Iran. Agric. For. Meteorol..

[31] Önöz B, Bayazit M (2003). The power of statistical tests for trend detection. Turkish J. Eng. Environ. Sci..

[32] Mozejko, J. Detecting and estimating trends of water quality parameters. in *Water Quality Monitoring and Assessment* (InTech, 2012). 10.5772/33052

[33] Helsel, D. R. *Nondetects and Data Analysis. Statistics for Censored Environmental Data. Nondetects and Data Analysis: Statistics for Censored Environmental Data* (Wiley-Interscience, 2005).

[34] da Silva RM (2015). Rainfall and river flow trends using Mann-Kendall and Sen’s slope estimator statistical tests in the Cobres River basin. Nat. Hazards.

[35] Krishnan V (2005). Probability and random processes. Probab. Random Process..

[36] Sen PK (1968). Estimates of the regression coefficient based on Kendall’s Tau. J. Am. Stat. Assoc..

[37] Fentaw, F., Melesse, A. M., Hailu, D. & Nigussie, A. Precipitation and streamflow variability in Tekeze River basin, Ethiopia. in *Extreme Hydrology and Climate Variability* 103–121 (Elsevier, 2019). 10.1016/B978-0-12-815998-9.00010-5

[38] Robinson EA (2013). Probability Theory and Applications.

[39] Ahsanullah, M., Kibria, B. M. G. & Shakil, M. *Normal and Student´s t Distributions and Their Applications*. *Atlantis Studies in Probability and Statistics* Vol 4 (Atlantis Press, 2014).

[40] IPCC. *Managing the Risks of Extreme Events and Disasters to Advance Climate Change Adaptation*. IPCC (2012). 10.1596/978-0-8213-8845-7 10.1136/jech-2012-201045 22766781

[41] Page E (1954). Continuous inspection schemes. Biometrika.

[42] Mansell, M. G. The effect of climate change on rainfall trends and flooding risk in the West of Scotland. *Hydrol. Res.* **28**, 37 LP–50 (1997).

[43] Krieter J, Engler J, Tölle KH, Timm HH, Hohls E (2009). Control charts applied to simulated sow herd datasets. Livest. Sci..

[44] Jhajharia D, Singh VP (2011). Trends in temperature, diurnal temperature range and sunshine duration in Northeast India. Int. J. Climatol..

[45] Dash SK, Sharma N, Pattnayak KC, Gao XJ, Shi Y (2012). Temperature and precipitation changes in the north-east India and their future projections. Glob. Planet. Change.

[46] Jain SK, Kumar V, Saharia M (2013). Analysis of rainfall and temperature trends in northeast India. Int. J. Climatol..

[47] Nandargi S, Dhar ON (2011). Extreme rainfall events over the Himalayas between 1871 and 2007. Hydrol. Sci. J..

[48] Shekhar MS, Chand H, Kumar S, Srinivasan K, Ganju A (2010). Climate-change studies in the western Himalaya. Ann. Glaciol..

[49] Borgaonkar HP, Pant GB (2001). Long-term climate variability over monsoon Asia as revealed by some proxy sources. Mausam.

[50] Kumar, V., Singh, P. & Jain, S. K. Rainfall trends over Himachal Pradesh, Western Himalaya, India. in *Development of Hydro Power Projects—A Prospective Challenge* 20–22 (2005).

[51] Basnett S, Kulkarni AV, Bolch T (2013). The influence of debris cover and glacial lakes on the recession of glaciers in Sikkim Himalaya, India. J. Glaciol..

[52] Racoviteanu, A. E., Arnaud, Y., Williams, M. W. & Manley, W. F. Spatial patterns in glacier characteristics and area changes from to in the Kanchenjunga-Sikkim area, eastern Himalaya. *Cryosph. 9 SRC*-**G**, 505–523, https://doi.org/10.5194tc9505 (2015).

[53] Debnath M, Sharma MC, Syiemlieh HJ (2019). Glacier dynamics in changme khangpu basin, sikkim himalaya, India, between 1975 and 2016. Geoscience.

[54] Debnath M (2018). Glacial lake dynamics and lake surface temperature assessment along the Kangchengayo-Pauhunri Massif, Sikkim Himalaya, 1988–2014. Remote Sens. Appl. Soc. Environ..

[55] Shukla A, Garg PK, Srivastava S (2018). Evolution of glacial and high-altitude lakes in the Sikkim, Eastern Himalaya over the past four decades (1975–2017). Front. Environ. Sci..

[56] Government of Sikkim. *Sikkim Action Plan on Climate Change (2012–2030).* (2011).

