# Supplementary material for: Climatic variability at Gangtok and Tadong weather observatories in Sikkim, India, during 1961–2017
Source: Sci Rep. 2020 Sep 16;10:15177. doi: 10.1038/s41598-020-71163-y (PMC7494871; doi:10.1038/s41598-020-71163-y)
Supplement: Supplementary file 1 — Supplementary information. [file 41598_2020_71163_MOESM1_ESM.docx]

**Climatic variability at Gangtok and Tadong weather observatories in Sikkim, India, during 1961-2017**

Parvendra Kumar^1,2, *^, Milap Chand Sharma^2^, Rakesh Saini^1^, Girish Kumar Singh^3^

^1, *^ Department of General & Applied Geography, Dr. Harisingh Gour Central University, Sagar, Madhya Pradesh, 470003, India.

^2^ Centre for the Study of Regional Development, Jawaharlal Nehru University, New Delhi, 110067, India.

^3^ Department of Computer Science, Dr. Harisingh Gour Central University, Sagar, Madhya Pradesh, 470003, India.

* **Corresponding author’s email.** [parvendra.jnu@gmail.com](mailto:parvendra.jnu@gmail.com)

**Contact no. +91 9479983931**

Supplementary Table S1. Trends of annual temperatures (˚C) for the overlapping period (1981-2010) at Gangtok using the Mann-Kendall trend test and Sen’s slope estimator.

|  | **Min.** | **Max.** | **Mean** | **Std.**  **deviation** | **CV (%)** | **Kendall's**  **tau** | **S** | **Var(S)** | **Z_MK_** | **p-value**  **(Two-tailed)** | **Alpha** | **Trend** | **Sen's**  **slope** |
| --- | --- | --- | --- | --- | --- | --- | --- | --- | --- | --- | --- | --- | --- |
| Max. Temp. | 17.71 | 20.90 | 18.66 | 0.48 | 2.57 | 0.22 | 98.00 | 3140..66 | 1.73 | 0.08 | 0.05 | N | 0.019 |
| Avg. Temp | 12.89 | 16.48 | 15.18 | 0.87 | 5.73 | 0.63 | 277.00 | 3140.66 | 4.92 | <0.0001 | 0.05 | Y+ | 0.066 |

Note: * indicates annual precipitation at the observed stations. Y+ indicates positive and N indicates no trend in the data.
